# Ca^2+^-Activated K^+^ Channels and the Regulation of the Uteroplacental Circulation

**DOI:** 10.3390/ijms24021349

**Published:** 2023-01-10

**Authors:** Xiang-Qun Hu, Lubo Zhang

**Affiliations:** Lawrence D. Longo, MD Center for Perinatal Biology, Department of Basic Sciences, School of Medicine, Loma Linda University, Loma Linda, CA 92350, USA

**Keywords:** Ca^2+^-activated K^+^ (K_Ca_) channels, uteroplacental circulation, vascular tone, estrogen, hypoxia, reactive oxygen species, endoplasmic reticulum stress, DNA methylation

## Abstract

Adequate uteroplacental blood supply is essential for the development and growth of the placenta and fetus during pregnancy. Aberrant uteroplacental perfusion is associated with pregnancy complications such as preeclampsia, fetal growth restriction (FGR), and gestational diabetes. The regulation of uteroplacental blood flow is thus vital to the well-being of the mother and fetus. Ca^2+^-activated K^+^ (K_Ca_) channels of small, intermediate, and large conductance participate in setting and regulating the resting membrane potential of vascular smooth muscle cells (VSMCs) and endothelial cells (ECs) and play a critical role in controlling vascular tone and blood pressure. K_Ca_ channels are important mediators of estrogen/pregnancy-induced adaptive changes in the uteroplacental circulation. Activation of the channels hyperpolarizes uteroplacental VSMCs/ECs, leading to attenuated vascular tone, blunted vasopressor responses, and increased uteroplacental blood flow. However, the regulation of uteroplacental vascular function by K_Ca_ channels is compromised in pregnancy complications. This review intends to provide a comprehensive overview of roles of K_Ca_ channels in the regulation of the uteroplacental circulation under physiological and pathophysiological conditions.

## 1. Introduction

Vascular tone in small arteries/arterioles governs vascular resistance and hence blood perfusion of a given tissue/organ. It is determined by the contractile state of vascular smooth muscle cells (VSMCs), which is regulated by dynamic changes in intracellular Ca^2+^ concentrations ([Ca^2+^]_i_) [1]. Vasoconstriction is initiated by an increase in [Ca^2+^]_i_ primarily due to Ca^2+^ influx mediated by the L-type voltage-dependent Ca^2+^ (Ca_V_1.2) channel in the plasma membrane and/or Ca^2+^ release mediated by ryanodine (RyR)/inositol trisphosphate (IP_3_R) receptors in the sarcoplasmic reticulum (SR) membrane. The activity of the Ca_V_1.2 channel in VSMCs is regulated by the membrane potential. Potassium (K^+^) channels are dominant ion conductive pathways in the vasculature to set/regulate the membrane potential. Their activities in endothelial cells (ECs) and VSMCs participate in regulating Ca^2+^ homeostasis and vascular tone [2,3]. Membrane hyperpolarization induced by the opening of K^+^ channels closes the Ca_V_1.2 channel in VSMCs, leading to a fall in [Ca^2+^]_i_ and subsequent vasodilatation. In contrast, membrane depolarization caused by the closing of K^+^ channels opens the Ca_V_1.2 channel, resulting in an increase in [Ca^2+^]_i_ and vasoconstriction. Therefore, the dynamic interplay between Ca_V_1.2 and K^+^ channels in the vasculature plays a pivotal role in regulating vascular tone. Among various types of K^+^ channels in ECs and VSMCs, Ca^2+^-activated K^+^ (K_Ca_) channels are instrumental in the regulation of vascular tone [2,3,4,5,6,7].

Uteroplacental blood flow increases dramatically during pregnancy. Adequate uteroplacental blood perfusion is essential for the growth/development of the placenta and fetus, as well as the well-being of the mother. Uteroplacental blood flow is inversely proportional to uteroplacental vascular resistance. Increased uteroplacental blood flow in pregnancy is primarily achieved by lowering uteroplacental vascular resistance owing to the structural remodeling of spiral arteries, establishment of the placenta, and vasodilation [8,9]. Adaptive changes in the uteroplacental circulation are impaired in pregnancy complications such as preeclampsia, fetal growth restriction (FGR, also known as intrauterine growth restriction), and gestational diabetes, leading to insufficient perfusion of the placenta [9,10,11]. These disorders are interrelated. For example, early-onset preeclampsia is often associated with FGR, whereas gestational diabetes is a risk factor for preeclampsia [12,13]. Pregnancy complications are associated with maternal and perinatal morbidity and mortality [14,15,16] and predispose the mother and offspring to metabolic and cardiovascular diseases in later life [17,18,19,20]. The first experimental evidence that K_Ca_ channels participate in regulating uteroplacental blood flow was presented by Rosenfeld’s group in 2000 [21]. Since then, molecular and functional expression of K_Ca_ channels in uteroplacental vessels has received considerable attention. In this review, we highlight current knowledge on the roles of K_Ca_ channels in the regulation of uteroplacental circulation in physiological and pathophysiological conditions.

## 2. Overview of K_Ca_ Channels

K_Ca_ channels are a large family of K^+^ channels, which are activated by intracellular Ca^2+^ and selectively transport K^+^ ions. K_Ca_ channels contain six/seven-transmembrane domains, and are classified into two groups based on their biophysical properties [22]. One group includes the BK_Ca_ channel that has large single-channel conductance ranging from 100 to 300 pS [23,24] and is activated by micromolar [Ca^2+^]_i_ and membrane depolarization [23,25]. The other group comprises small-conductance (SK_Ca_) (K_Ca_2.1–2.3) and intermediate-conductance (IK_Ca_, K_Ca_3.1) K_Ca_ channels that are voltage-insensitive and are activated by sub-micromolar [Ca^2+^]_i_. The SK_Ca_ channel has single-channel conductance of 5–20 pS [26,27], whereas the IK_Ca_ channel has unitary conductance of 20–40 pS [28,29].

**Figure 1:**
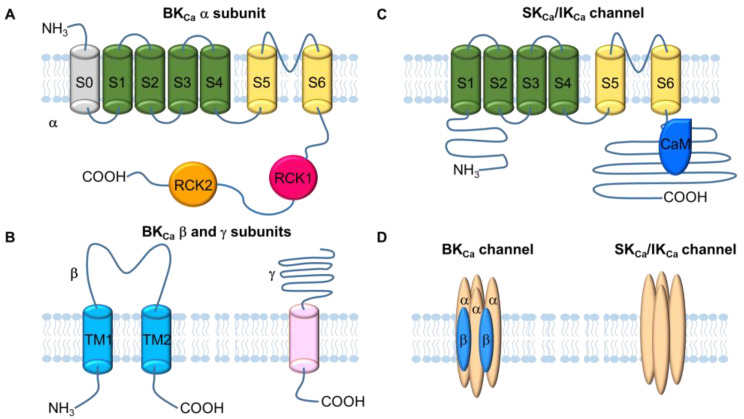
Ca^2+^-activated K^+^ (K_Ca_) channel topology and structure. (**A**) Membrane topology of the BK_Ca_ channel α subunit. The α subunit contains the S0 segment, voltage sensing domain (S1–S4 segments), pore gate domain (S5 and S6 segments), and cytosolic domain containing RCK1 and RCK2. Ca^2+^ sensitivity is conferred by binding of Ca^2+^ to RCK1 and RCK2. (**B**) Membrane topology of BK_Ca_ channel β and γ subunits. The β subunit is comprised of two transmembrane domains, whereas the γ subunit possesses only one transmembrane domain with six leucine-rich repeat segments in its extracellular domain. (**C**) Membrane topology of SK_Ca_ and IK_Ca_ channels. Both SK_Ca_ and IK_Ca_ channels contain six transmembrane domains. Ca^2+^ sensitivity is conferred by constitutively bound calmodulin (CaM) to the intracellular C-terminus. (**D**) K_Ca_ channels are either heterotetrameric (BK_Ca_) or homotetrameric (SK_Ca_ and IK_Ca_) assemblies of subunits.

A functional BK_Ca_ channel is composed of a tetramer of α-subunit that is encoded by the KCNMA1 gene. The BK_Ca_ channel achieves its functional diversity primarily through the association of α subunits with accessory subunits and other proteins, alternative splicing, and post-translational modifications such as phosphorylation, oxidation, and palmitoylation [30,31,32,33,34,35,36,37]. Each BK_Ca_ channel α subunit (125–140 kDa) contains seven transmembrane spanning segments (S0–S6) and a large cytoplasmic COOH-terminus (Figure 1). They form three main structural domains that serve distinct functions [38]. S1-S4 segments constitute the voltage-sensing domain that detects changes in the membrane potential. S5–S6 segments line the pore to control K^+^ permeation [39,40]. Two tandem RCK (regulator of conductance for K^+^) domains (RCK1 and RCK2) in the cytoplasmic COOH-terminus from each subunit form a Ca^2+^ gating ring and function as a Ca^2+^ sensor [41].

The BK_Ca_ channel is ubiquitously distributed among mammalian tissues [39] and usually associates with auxiliary β-subunits (~20 kDa). These accessory proteins are expressed in a cell-specific manner and display unique regulatory effects on the channel. Four distinct β-subunits, β1–4, are encoded by KCNMB1-4 [22]. The β1 subunit is primarily expressed in smooth muscle [42], whereas β2, β3, and β4 subunits are mostly expressed in neurons, chromaffin cells, kidney, heart, liver, and lung, among others [43,44,45]. The β-subunit consists of two transmembrane domains with intracellular N- and C-termini and a long extracellular loop (Figure 1). Up to four β-subunits could co-assemble with pore-forming α subunits [46,47]. Co-assembling with these auxiliary subunits alters the channel’s apparent sensitivity to Ca^2+^ and voltage as well as kinetic properties [35].

A group of leucine-rich repeat-containing (LRRC) proteins (~35 kDa) are identified as auxiliary γ subunits of the BK_Ca_ channel [48]. The expression of LRRC proteins is also tissue-dependent [49]. These LRRC proteins are structurally distinct from the β-subunit. They consist of a large, extracellular domain with six leucine-rich repeat units (LRR1–6), and a single transmembrane segment (Figure 1). In a manner similar to the β subunit, the association of γ subunits to α subunits also alters channel gating properties by increasing voltage sensitivity even in the absence of Ca^2+^ [35].

SK_Ca_ channels are encoded by KCNN1-3, whereas the IK_Ca_ channel is encoded by KCNN4. SK_Ca_ and IK_Ca_ channels share a similar topology to members of the K_V_ channel superfamily and consist of six transmembrane segments (S1–S6) [50] (Figure 1). They are also tetrameric structures. The channel pore is formed by S5 and S6. However, the S4 segment of SK_Ca_ and IK_Ca_ channels contains fewer charged residues than its counterparts in the K_V_ and BK_Ca_ channels, resulting in a lack of voltage dependence. These channels are expressed primarily in neurons and ECs. Although the activities of SK_Ca_ and IK_Ca_ channels are also controlled by intracellular Ca^2+^ levels, Ca^2+^ does not directly bind to channels. Instead, the Ca^2+^ sensitivity of these channels is achieved through the binding of Ca^2+^ to calmodulin (CaM) constitutively bound to the C-terminus of the channel [51,52].

## 3. K_Ca_ Channels and Vascular Function

### 3.1. K_Ca_ Channels in VSMCs

The BK_Ca_ channel α subunit is abundantly expressed in VSMCs of virtually all vascular beds. BK_Ca_ channel accessory β and γ subunits are also found in VSMCs [46,53,54,55]. The predominant β isoform in VSMCs is the β1 subunit [42]. Although β2 and β4 subunits are also present in VSMCs of some vessels, their expression is extremely low [56,57,58]. The association of accessory subunits with α subunits alters channel biophysical properties. Both β1 and γ subunits increase BK_Ca_ channel sensitivity to both Ca^2+^ and voltage in VSMCs [42,53].

SK_Ca_ channels are scantily expressed in VSMCs [59,60]. Although an apamin-sensitive K^+^ conductance has been demonstrated in VSMCs of some vascular beds [61,62,63], its identity has not been resolved. Similarly, evidence for the existence of IK_Ca_ channels in VSMCs is limited. The IK_Ca_ channel is either not or very poorly expressed in contractile VSMCs [64]. However, its expression in VSMCs is significantly upregulated during proliferation or under pathophysiological conditions such as myocardial infarction, vascular injury, and atherosclerosis [29,64,65,66,67]. Therefore, the IK_Ca_ channel likely plays a role in the angiogenesis and pathogenesis of atherosclerosis/restenosis. However, SK_Ca_ and IK_Ca_ channels are found to express in VSMCs of uterine and placental chorionic plate arteries [68,69].

### 3.2. K_Ca_ Channels in ECs

Endothelial expression of the BK_Ca_ channel appears to be erratic [70,71]. Molecular expression of the BK_Ca_ channel α subunit and channel activity have been reported in the intact endothelium and in isolated ECs from some blood vessels [72,73,74,75,76,77,78]. However, the BK_Ca_ channel is absent in ECs from other vascular beds [79,80,81,82,83,84]. In addition, the BK_Ca_ channel β1 subunit is absent in ECs [77,85]. Proliferation and chronic hypoxia trigger BK_Ca_ channel expression in ECs [86,87,88]. Of interest, the BK_Ca_ channel β4 subunit along with the α subunit is expressed in rat lung microvascular ECs, forming functional BK_Ca_ channels [77].

Both IK_Ca_ and SK_Ca_ channels are abundantly expressed in the endothelium [2,89]. The predominant SK_Ca_ and IK_Ca_ channels expressed in ECs are K_Ca_2.3 (SK3) and K_Ca_3.1 (IK1) channels, respectively [90,91]. It appears that SK_Ca_ and IK_Ca_ channels have distinct spatial localizations. Whereas K_Ca_2.3 channels are widely distributed in the EC plasma membrane, K_Ca_3.1 channels are primarily located in myoendothelial gap junctions (MEGJs) [92,93].

### 3.3. K_Ca_ Channels and the Regulation of Vascular Function

#### 3.3.1. Activation of BK_Ca_ Channels in VSMCs

**Figure 2:**
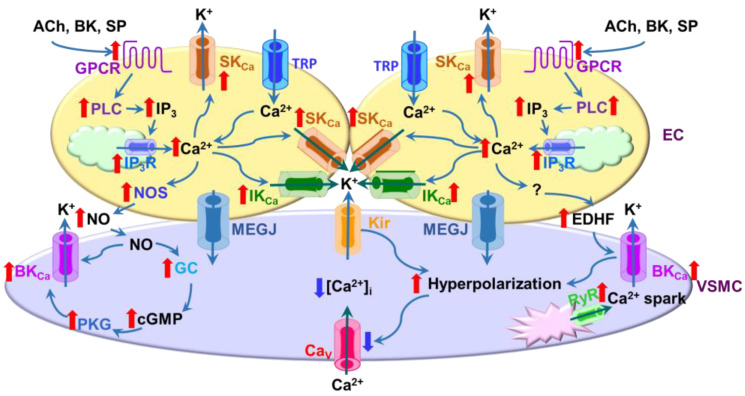
Regulation of vascular function by cross-talks among ion channels. The BK_Ca_ channel is preferentially expressed in vascular smooth muscle cells (VSMCs), whereas SK_Ca_ and IK_Ca_ channels are primarily expressed in endothelial cells (ECs). Vasoconstriction is triggered by an increase in intracellular Ca^2+^ ([Ca^2+^]_i_) in VSMCs due to Ca^2+^ release from the sarcoplasmic reticulum (SR) and/or Ca^2+^ influx through the Ca_V_ 1.2 channel in the plasma membrane. Vasoconstriction is counteracted by activities of SK_Ca_/IK_Ca_ channels in ECs and BK_Ca_ channels in VSMCs. The activation of SK_Ca_ and IK_Ca_ channels in ECs triggered by inositol triphosphate receptor (IP_3_R)-mediated Ca^2+^ release and/or transient receptor potential (TRP) channel-mediated Ca^2+^ influx promotes hyperpolarization and release of nitric oxide (NO)/endothelium-derived hyperpolarizing factor (EDRF). In addition, hyperpolarization in ECs could be transmitted to VSMCs via myoendothelial gag junctions (MEGJs). The BK_Ca_ channel is activated by RyR-mediated Ca^2+^ sparks in VSMCs. BK_Ca_ channel activity is also subject to regulation by EC-derived NO and EDHF. Moreover, the accumulation of K^+^ ions in the intercellular space hyperpolarizes VSMCs by activating the inwardly rectifying K^+^ (K_ir_) channel. Overall, these events promote hyperpolarization of VSMCs, which in turn leads to the closure of the Ca_V_ 1.2 channel and subsequent vasodilation. ACh, acetylcholine; BK, bradykinin; SP, substance P; GPCR, G protein-coupled receptor; PLC, phospholipase C; NOS, nitric oxide synthases; GC, guanylate cyclase; cGMP, cyclic guanosine monophosphate; PKG, protein kinase G.

Given the large conductance and copious expression of the BK_Ca_ channel in VSMCs, small changes in the open probability of the channel have a significant impact on the membrane potential of VSMCs and vascular tone. BK_Ca_ channel activation in VSMCs is primarily linked to Ca^2+^ release events from the SR through RYRs and/or Ca^2+^ influx through Ca_V_1.2 channels or nonselective cation ion channels (Figure 2) [94,95,96]. A fraction of RYRs in the SR membrane are in close proximity to BK_Ca_ channels in the plasma membrane of VSMCs and together they form Ca^2+^ signaling microdomains [97]. Concerted opening of several RyRs generates Ca^2+^ sparks and the local [Ca^2+^]_i_ may reach ~10 μM within these microdomains [97,98,99]. Ca^2+^ sparks then activate BK_Ca_ channels to produce spontaneous transient outward currents (STOCs), which in turn promote membrane hyperpolarization and closure of the Ca_V_1.2 channel. The BK_Ca_ channel β1 subunit plays a central role in linking Ca^2+^ sparks to the BK_Ca_ channel. Genetic deletion of the β1 subunit decreases the Ca^2+^ sensitivity of the BK_Ca_ channel, resulting in uncoupling BK_Ca_ channels from Ca^2+^ sparks [100]. In addition, reduced expression of the BK_Ca_ channel β1 subunit in type 2 diabetic murine VSMCs leads to abnormal coupling between Ca^2+^ sparks and the BK_Ca_ channel [101]. Ca_V_1.2, BK_Ca_, and transient receptor potential canonical 1 (TRPC1) channels can form complexes in the plasma membrane of VSMCs to provide an efficient mechanism for obtaining localized high Ca^2+^ concentrations to activate the BK_Ca_ channel [102,103,104,105]. Additionally, TRPV4, RyRs, and BK_Ca_ channels are also found to form Ca^2+^ signaling complexes to promote smooth muscle hyperpolarization [95]. Furthermore, the generation of Ca^2+^ sparks can be indirectly modulated by the Ca_V_1.2 channel_._ Ca_V_1.2 channel-mediated Ca^2+^ entry increases luminal SR Ca^2+^ and hence Ca^2+^ sparks [106]. Thus, the formation of Ca^2+^ microdomains/macromolecular complexes provides a rapid feedback and elicits an efficient regulation of Ca^2+^ signaling in VSMCs.

#### 3.3.2. BK_Ca_ Channels and Vascular Tone

VSMCs of small arteries/arterioles possess intrinsic properties to constrict in response to an increase in intralumenal pressure and to dilate following a decrease in intralumenal pressure [107]. An increase in intralumenal pressure depolarizes the plasma membrane leading to the opening of the Ca_V_1.2 channel and vasoconstriction/myogenic tone. However, myogenic vasoconstriction is regulated by a negative feedback mechanism conferred by the BK_Ca_ channel [5]. Membrane depolarization promotes Ca^2+^ sparks in VSMCs. In addition, Ca^2+^ entry through Ca_V_1.2 and TRPV4 channels also enhances Ca^2+^ sparks that in turn activate the BK_Ca_ channel [96]. Activation of the BK_Ca_ channel in VSMCs triggers STOCs and subsequent membrane hyperpolarization, leading to Ca_V_1.2 channel closure and vasodilation [94]. Therefore, the BK_Ca_ channel functions as a ‘brake’ to prevent excessive vasoconstriction. The importance of the BK_Ca_ channel in the regulation of vascular function has been well demonstrated by pharmacological and genetic manipulations. The blockade of the BK_Ca_ channel with iberiotoxin or tetraethylammonium (TEA) induces membrane depolarization, followed by an elevation of [Ca^2+^]_i_, vasoconstriction, and elevated blood pressure [42,108,109,110]. Genetic ablation of the BK_Ca_ channel α subunit leads to hypertension [111], suggesting an essential role of this channel in regulating blood pressure and controlling blood perfusion to organs. The BK_Ca_ channel β1 subunit is also vital in regulating vascular tone. The BK_Ca_ channel in VSMCs from β1 null mice has decreased Ca^2+^ sensitivity and reduced channel activity due to uncoupling the channel from Ca^2+^ sparks. These changes result in VSMC membrane depolarization and enhancement of vasoconstriction, which ultimately lead to the development of hypertension [42,100,112,113]. Not surprisingly, the expression of the BK_Ca_ channel β1 subunit in VSMCs is reduced in hypertension in patients [114] and in animal models [115,116,117]. In contrast, a gain-of-function mutation of the BK_Ca_ channel β1 subunit is associated with a low prevalence of hypertension in human studies [118,119,120]. In addition, the expression of the BK_Ca_ channel β1 subunit in VSMCs of rat mesenteric arteries is upregulated after hemorrhagic shock [121]. This upregulation enhances Ca^2+^ sensitivity of the BK_Ca_ channel, promotes VSMC membrane hyperpolarization, and reduces vasoconstriction to norepinephrine. Diabetes is also associated with suppressed expression of the BK_Ca_ channel β1 subunit in VSMCs [122,123].

The BK_Ca_ channel activity is fine-tuned by phosphorylation [37,124]. Many vasoactive agents alter vascular contractility via protein kinase-mediated phosphorylation of the BK_Ca_ channel. Endothelin, angiotensin II, 5-hydroxytryptamine, and 20-hydroxyeicosatetraenoic acid elicit vasoconstriction via serine/threonine kinase PKC- and/or tyrosine kinase c-Src-mediated inhibition of the BK_Ca_ channel in VSMCs [125,126,127,128,129]. Conversely, β-adrenergic agonists, adenosine, calcitonin gene-related peptide, and nitric oxide (NO) mainly produce vasorelaxation via PKA- or PKG-dependent activation of the BK_Ca_ channel in VSMCs [130,131,132,133,134,135,136].

NO can also regulate BK_Ca_ channel activity in VSMCs by altering the trafficking of the BK_Ca_ channel β1 subunit. NO is found to stimulate rapid surface trafficking of the BK_Ca_ channel β1 subunit via cGMP-PKG- and cAMP-PKA-dependent pathways, resulting in increased channel Ca^2+^ sensitivity/channel activity, and vasodilation [137]. Moreover, NO is able to directly activate the BK_Ca_ channel in VSMCs [138,139].

#### 3.3.3. Activation of SK_Ca_ and IK_Ca_ Channels in ECs

The vascular endothelium plays a key role in regulating vascular tone. Activation of SK_Ca_ and IK_Ca_ channels is an essential process for endothelium-dependent vasorelaxation conferred by various vasoactive agents [60,81,140,141,142,143]. Endothelium-dependent vasodilators and physical stimuli such as fluid shear stress increase [Ca^2+^]_i_ in ECs by triggering IP_3_R-mediated Ca^2+^ release from SR, store-operated Ca^2+^ entry, and TRPV4-mediated Ca^2+^ influx [144]. Ca^2+^ subsequently binds to calmodulin constitutively bound to SK_Ca_ and IK_Ca_ channels, resulting in channel conformational changes and channel activation [145].

#### 3.3.4. SK_Ca_ and IK_Ca_ Channels and Vascular Tone

Opening endothelial SK_Ca_ and IK_Ca_ channels induces hyperpolarization, which could be transmitted to adjacent VSMCs via MEGJ, leading to hyperpolarization of VSMCs, closure of the Ca_V_1.2 channel, and subsequent vasodilation (Figure 2) [2,146,147,148]. In addition, K^+^ ion accumulated in the extracellular space between ECs and VSMCs due to activation of endothelial SK_Ca_ and IK_Ca_ channels is proposed to cause hyperpolarization and relaxation of the VSMCs through activating the inwardly-rectifying K^+^ (K_ir_) channel and/or the Na^+^-K^+^-ATPase [149,150]. Furthermore, both SK_Ca_ and IK_Ca_ channels also participate in regulating NO synthesis and release from ECs [151,152,153]. The blockade of the SK_Ca_ channel with apamin and of the IK_Ca_ channel with charybdotoxin or triarylmethane-34 (TRAM-34) attenuates NO production in ECs [151,152]. Activation of endothelial SK_Ca_ and IK_Ca_ channels also promotes the release of endothelium-derived hyperpolarizing factor (EDHF) [154]. Depending on the size of the vessels, different mechanisms may be involved in the actions of SK_Ca_ and IK_Ca_ channels. Activating endothelial SK_Ca_ and IK_Ca_ channels causes vasorelaxation mainly via the release of NO in large arteries and EDHFs in small arteries, respectively [155,156]. NO and EDHFs released from ECs subsequently trigger BK_Ca_ channel activation in VSMCs, leading to vasorelaxation [139,157,158,159]. Pharmacologic blockade or genetic ablation of SK_Ca_ and/or IK_Ca_ channels depolarizes ECs and decreases vasoactive agent-evoked hyperpolarization of ECs and VSMCs, resulting in impaired vasorelaxation and reduced blood flow [59,151,152,160,161,162,163,164]. Conversely, SK_Ca_ and IK_Ca_ channel activation decreases vascular tone/blood pressure and increases blood flow [153,163,165,166,167]. The functional importance of SK_Ca_ and IK_Ca_ channels is furthermore supported by observations that deletion of either or both SK_Ca_ and IK_Ca_ genes is associated with the development of hypertension [59,164,168]. Consistent with these findings, the expression of SK_Ca_2.3 and/or IK_Ca_ channels was reduced in mesenteric arteries from spontaneously or ANG II-induced hypertensive rats [169,170]. However, the IK_Ca_ channel is upregulated under certain pathophysiological conditions such as myocardial infarction, and atherosclerosis [64,171,172,173]. In addition, the expression of SK_Ca_2.3 and IK_Ca_ channels is differently altered by chronic hypoxia in pulmonary arteries. Exposure to chronic hypoxia causes upregulation of the SK_Ca_2.3 channel, but downregulation of the IK_Ca_ channel [174].

## 4. Adaptation/Maladaptation of the Uteroplacental Circulation in Normal Pregnancy and Pregnancy Complications

In a nonpregnant state, blood flow to the uterus is relatively low. For example, uterine blood flow is ~20–50 mL/min in nonpregnant humans and sheep, corresponding to 1–3% of the maternal cardiac output [175,176,177,178]. Uteroplacental blood flow increases dramatically during pregnancy, rising to 600 to 1000 mL/min at 36 to 38 weeks in human pregnancy [179,180] and >1000 mL/min in late sheep pregnancy [175,181,182,183]. Similarly, uteroplacental blood flow increases by 10- to 30-fold in near-term pregnant rats and guinea pigs [184,185,186]. Uteroplacental blood flow comprises ~20% of maternal cardiac output at term [179,186,187]. It is estimated that 80% to 90% of total uteroplacental blood flow perfuses the placenta at term and the remaining supplies the myometrium [175,188,189], providing sufficient nutrient and oxygen supply for the growth of the placenta and fetus. The hemodynamic changes in the uteroplacental circulation during pregnancy are primarily achieved by uterine vascular remodeling, reduced uteroplacental vascular resistance, and the formation of the placenta [8,9,190,191,192]. Notably, a variety of functional changes contribute to the adaptation. Myogenic tone is markedly attenuated in the uterine arteries of pregnant sheep [193]. Vasopressor response of uterine arteries to various vasoconstrictors such as α-adrenergic agonists, 5-hydroxytryptamine, endothelin 1, angiotensin II, and thromboxane is attenuated during pregnancy in humans and other species [194,195,196,197,198,199,200,201,202]. Moreover, the production of vasodilators including NO and EDHF in uterine arteries increases during pregnancy [203,204]. NO- and endothelium-dependent vasodilation in uterine arteries is also enhanced during pregnancy [203,204,205,206,207,208].

The adaptation of the uteroplacental circulation is compromised in preeclampsia, FGR, and gestational diabetes. Preeclampsia is associated with increased uteroplacental vascular resistance [209,210,211]. Uterine arteries from preeclamptic women and animal models of preeclampsia display enhanced vasoconstriction and blunted vasodilation to vasoactive agents [212,213,214,215,216,217,218]. In addition, shear stress-mediated NO release from uterine arterial endothelium is impaired in preeclampsia [219]. EDHF-mediated vasorelaxation of myometrial arteries is reduced in preeclampsia [214,220]. In a rat model of preeclampsia produced by reduced uterine perfusion pressure (RUPP) in pregnant animals, uterine arteries exhibit increased myogenic tone and decreased endothelium-dependent vasorelaxation [221]. Additionally, the refractoriness to angiotensin II in uterine arteries is lost in gestational hypertension [222,223]. Uteroplacental vascular resistance is increased in a mouse model of gestational diabetes [224]. Endothelium-dependent vasorelaxation is impaired in the myometrial arteries of women with diabetes [225]. As expected, uteroplacental blood flow is reduced in preeclampsia, FGR, and gestational diabetes [210,226,227,228,229].

## 5. K_Ca_ Channels and the Uteroplacental Circulation in Normal Pregnancy

### 5.1. K_Ca_ Channels in Uteroplacental Vasculature

Both real-time polymerase chain reaction (RT-PCR) and Western blot reveal the expression of BK_Ca_ channel α, β1, and β2 subunits in the uterine arteries of humans and sheep [21,57,230,231,232,233,234,235]. The β1 subunit is the predominant β isoform in uterine arteries, and the expression level of the β2 subunit is low. Immunohistochemistry further reveals that these BK_Ca_ channel subunits are located in VSMCs, but not in the endothelium, of uterine arteries [57,230,231,232]. The BK_Ca_ channel in VSMCs of uterine arteries is activated by an increase in [Ca^2+^]_i_, and has unitary conductance of 100–200 pS [21,236]. The BK_Ca_ channel γ subunit is also detected in both human and mouse uterine arteries [55,236]. SK_Ca_ and IK_Ca_ channels are also expressed in uterine arteries [68,237]. IK_Ca_ channel mRNA is detected in cultured human uterine microvascular ECs [238]. Both SK_Ca_ and IK_Ca_ channels have been visualized in the endothelium of human and sheep uterine arteries with immunohistochemistry [68,239]. Of interest, K_Ca_2.2 and K_Ca_2.3 channels are present in VSMCs of sheep uterine arteries [68]. BK_Ca_, IK_Ca_, and K_Ca_2.3 channels are also detected in VSMCs and/or ECs of placental chorionic plate arteries of pregnant women [240,241].

### 5.2. K_Ca_ Channels in the Adaptation of the Uteroplacental Circulation in Normal Pregnancy

#### 5.2.1. Estrogen as a Key Determinant of K_Ca_ Channel Upregulation

The expression of K_Ca_ channels in uteroplacental vessels is under the influence of estrogen during the ovarian cycle and pregnancy. Khan et al. demonstrate that the BK_Ca_ channel α subunit protein in ovine uterine arteries remains constant during both follicular and luteal phases of the ovarian cycle [232]. The protein level of the BK_Ca_ channel β1 subunit is higher in uterine arteries from follicular phase ewes than in vessels from luteal phase animals. Similarly, protein abundance of the BK_Ca_ channel α subunit in uterine arteries is negligibly affected by gestation, whereas the expression of the BK_Ca_ channel β1 subunit is upregulated in uterine arteries from pregnant sheep [57,233]. The upregulation of the BK_Ca_ channel β1 subunit expression in uterine arteries during the follicular phase of the ovarian cycle and during pregnancy is paralleling with elevated plasma estrogen levels [57,232]. Remarkably, prolonged treatment of nonpregnant sheep or isolated uterine arteries from nonpregnant animals with 17β-estradiol increases the BK_Ca_ channel β1 subunit expression in the uterine vasculature, resembling those changes that occurred during the ovarian cycle and gestation [230,233,235]. Similarly, estrogen treatment and pregnancy also increase BK_Ca_ channel β1 subunit expression in rat uterus [242]. These observations implicate estrogen as an initiator for the upregulation of BK_Ca_ channel expression in the uterus and its vascular beds in pregnancy. The expression of the BK_Ca_ channel β2 subunit in uterine arteries remains low and unchanged during pregnancy [57]. The increased expression of the BK_Ca_ channel β1 subunit alters channel stoichiometry and increases Ca^2+^ sensitivity. In addition, pregnancy and prolonged treatment of nonpregnant sheep with 17β-estradiol also upregulate the expression of NOS, PKG-1α, and cGMP in uterine arteries [57,230,232,243,244]. The upregulation of the NO-cGMP-cPKG pathway could stimulate the BK_Ca_ channel through phosphorylation [245]. The enhanced BK_Ca_ channel activity subsequently contributes to reduced uterine vascular resistance [233].

Pregnancy also upregulates SK_Ca_ channel expression in uterine arteries [68]. This upregulation is also simulated by ex vivo estrogen treatment of isolated uterine arteries from nonpregnant sheep. The expression of K_Ca_2.3 and IK_Ca_ channels in the aorta is increased in pregnant mice [246]. Similarly, estrogen replacement in ovariectomized rats increases the K_Ca_2.3 channel expression in the uterus and nonvascular smooth muscle [247,248]. In contrast, ovariectomy reduces K_Ca_2.3 channel activity and endothelium-dependent vasorelaxation in mouse mesenteric arteries [249]. Likewise, incubating human uterine microvascular ECs with high concentrations of estrogen or serum from normal pregnant women promotes SK_Ca_2.3 and IK_Ca_ channel expression [246]. Moreover, the treatment with serum from normal pregnant women increases plasma membrane abundance of SK_Ca_2.3 and IK_Ca_ channels in human uterine microvascular ECs [250]. As expected, estrogen replacement in ovariectomized rats enhances EDHF-mediated vasodilation of uterine arteries [251]. However, estrogen replacement in ovariectomized mice reduces K_Ca_2.3 channel expression in the uterus [252].

#### 5.2.2. Mechanisms Underlying Estrogen-Mediated K_Ca_ Channel Upregulation

Estrogen usually regulates gene expression via interacting with its classical receptors, ERα and ERβ. The binding of estrogen results in conformational changes of estrogen receptors, allowing these receptors to interact with estrogen response elements (EREs) in the promoter region of target genes to regulate transcription [253]. However, examination of the cloned ovine KCNMB1 promoter sequences reveals that this promoter contains no EREs [235]. Instead, ERα interacts with Sp1 and binds to Sp1 binding sites to regulate KCNMB1 expression in ovine uterine arteries. Several putative transcription factor binding sites, containing CpG dinucleotides in or near their core binding sequences, have been identified in ovine KCNMB1 promoter, including Sp1 at −380 and AP1 at −652, −879, and −1202. Among these sites, the Sp1_-380_ binding element is essential for ovine KCNMB1 gene expression as deletion of this site significantly decreases the KCNMB1 promoter activity [235]. The importance of Sp1 in the regulation of expression of KCNMB1 is also demonstrated in nonvascular smooth muscle. Overexpression of Sp1 in smooth muscle cells of rabbit sphincter of Oddi enhances KCNMB1 promoter activity [254].

**Figure 3:**
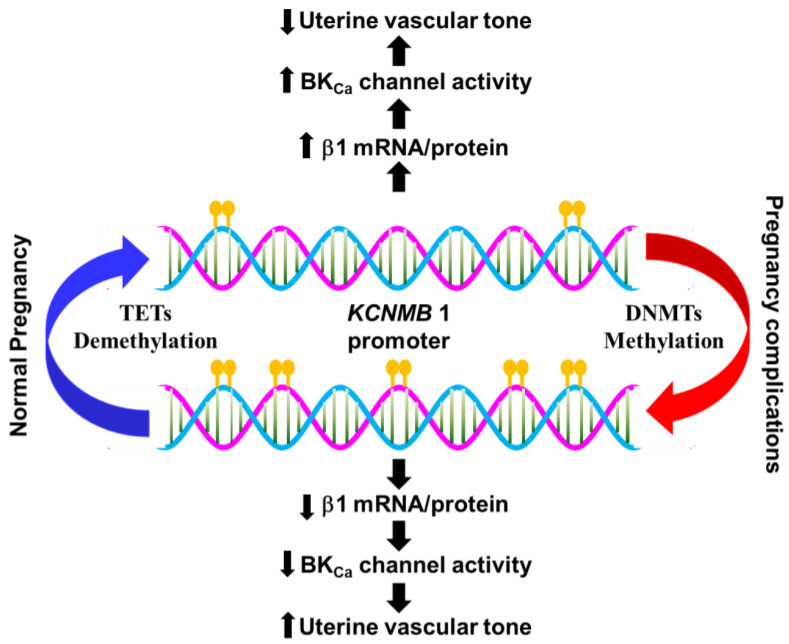
Epigenetic regulation of BK_Ca_ channel β1 subunit expression in uterine vasculature. The expression of the BK_Ca_ channel β1 subunit in uterine arteries of nonpregnant sheep is low due to hypermethylation of the KCNMB 1 promoter. Pregnancy increases the expression of the BK_Ca_ channel β1 subunit via promoting TET1-mediated demethylation of the promoter. Gestational hypoxia promotes methylation by upregulating DNMT3b and suppresses demethylation by downregulating TET1, leading to KCNMB 1 repression.

DNA methylation, the covalent addition of a methyl group (-CH3) to the base cytosine in the dinucleotide 5′-CpG-3′ catalyzed by DNA methyltransferases (DNMTs), is an important epigenetic mechanism controlling gene expression [255]. DNA methylation is usually associated with gene repression. CpG dinucleotides of the Sp1 binding site at the KCNMB1 gene promoter are highly methylated in the uterine arteries of nonpregnant sheep, resulting in low transcription factor binding and KCNMB1 promoter activity. Ten-eleven translocation methylcytosine dioxygenases (TETs) catalyze the conversion of 5-methylcytosine (5mC) to 5-hydroxymethylcytosine (5hmC) in active DNA demethylation. Pregnancy via estrogen upregulates TET1 which in turn decreases CpG methylation at the Sp1 binding site and facilitates Sp1/ERα binding to the Sp1 binding site of KCNMB1, leading to the upregulation of the BK_Ca_ channel β1 subunit in uterine arteries [235,256] (Figure 3).

The increased SK_Ca_ channel expression in uterine arteries during pregnancy is also mediated by estrogen [68]. Estrogen regulates SK_Ca_2.3 gene (KCNN3) expression through interactions between ERα and Sp1 in Cos7 and L6 cells [257]. Moreover, estrogen treatment stimulates the expression of the SK_Ca_2.3 transcript in human myometrial cells overexpressing Sp1 [252]. These observations suggest an important role of Sp1 in the expression of the KCNN3 gene.

Vascular endothelial growth factor (VEGF) appears to play role in the pregnancy-induced upregulation of SK_Ca_2.3 and IK_Ca_ channels. The upregulation of SK_Ca_2.3 and IK_Ca_ channels induced by exposure to serum from normal pregnant women in cultured human uterine microvascular ECs is diminished by blocking VEGF receptors [246]. Serum from normal pregnant women and VEGF increases H_2_O_2_ generation and promote SK_Ca_2.3 and IK_Ca_ channel expression via the H_2_O_2_/FYN/ERK pathway [246]. VEGF receptor activation also causes the downregulation of caveolin-1 and subsequently inhibits the internalization of SK_Ca_2.3 and IK_Ca_ channels, leading to their high abundance in the plasma membrane in uterine vascular ECs in pregnancy [250]. It should be noted that placental VEGF expression is also subject to regulation by estrogen in pregnancy [258].

#### 5.2.3. K_Ca_ Channels and the Adaptation of the Uteroplacental Circulation

Findings from in vivo and in vitro studies exploring the functional roles of K_Ca_ channels in the uterine circulation of nonpregnant sheep are quite intriguing. Despite the expression of the BK_Ca_ channel in uterine arteries of nonpregnant animals, stimulation of the BK_Ca_ channel with NS 1619 fails to promote vasorelaxation of these vessels [68,259]. In addition, the blockade of the BK_Ca_ channel with TEA also does not alter the myogenic tone of uterine arteries [233]. Moreover, basal uterine blood flow in nonpregnant sheep is negligibly altered by local infusion of TEA [21]. These findings suggest that the BK_Ca_ channel in the uterine arteries of nonpregnant sheep is quiescent and contributes minimally to the regulation of uterine vascular tone, vascular reactivity, and basal uterine blood flow. Interestingly, pregnancy ‘awakes’ the BK_Ca_ channel and the channel becomes active in ovine uterine arteries. Activation of the BK_Ca_ channel promotes vasorelaxation of uterine arteries from pregnant sheep [68,259], whereas inhibition of the BK_Ca_ channel increases the myogenic tone of uterine arteries [233]. Moreover, local infusion of TEA into uterine arteries decreases basal uterine blood flow by ~50% in pregnant sheep [182,260].

It is currently unknown why the BK_Ca_ channel is dormant in the uterine arteries of nonpregnant sheep. One possible explanation is the low abundance of the channel in uterine arteries. The other scenario is that the majority of the BK_Ca_ channel β1 subunit in uterine arteries of nonpregnant sheep are in the cytoplasm and do not form complexes with the α subunit at the surface membrane as observed in rat mesenteric and human cerebral arteries [137]. Leo et al. [137] demonstrate that NO stimulates rapid trafficking of the BK_Ca_ channel β1 subunit to the plasma membrane via a PKG-dependent pathway. Pregnancy is accompanied by parallel increases in NO, cGMP, protein kinase G-1α and the BK_Ca_ channel β1 subunit in uterine arteries [57,203,233]. We recently demonstrated that pregnancy increases the association of α and β1 subunits in uterine arteries [261]. The association of BK_Ca_ channel β1 and α subunits has been shown to increase channel activity by enhancing the channel’s Ca^2+^ sensitivity [42]. It is reasonable to speculate that the enhanced NO-PKG pathway in uterine arteries could stimulate the trafficking of the BK_Ca_ channel β1 subunit to the plasma membrane of VSMCs in addition to increased BK_Ca_ channel β1 subunit expression, thus facilitating the transition from the dormant BK_Ca_ channel in the nonpregnant state to the active channel in pregnancy.

BK_Ca_ channel activity is subject to modulation by protein kinases [37,124]. Activation of protein kinase C inhibits the BK_Ca_ channel in uterine arteries [233,262]. Thus, vasoconstriction induced by α-adrenergic ligands and thromboxane may involve PKC-mediated inhibition of the BK_Ca_ channel in this vessel [263,264]. Notably, PKC activity in uterine arteries is suppressed in pregnancy [193,264,265]. On the other hand, the production of vasodilators such as NO, calcitonin gene-related peptide, and adrenomedullin is increased in pregnancy and they produce vasorelaxation of uterine arteries apparently via cGMP-mediated activation of the BK_Ca_ channel [231,266,267]. Inhibition of the BK_Ca_ channel enhances uterine vasoconstriction induced by α-adrenergic ligands, thromboxane, and PKC activator in intact sheep and in isolated vessels [231,262,268,269]. Therefore, activation of the BK_Ca_ channel could offset vasoconstriction and prevents vasospasm of uterine arteries, which probably contributes to the refractoriness of uterine arteries to vasoconstrictors during normal pregnancy.

In VSMCs, the BK_Ca_ channel is primarily activated by Ca^2+^ sparks mediated by RyRs [270]. Activated BK_Ca_ channels then mediate K^+^ efflux in the form of STOCs, leading to membrane hyperpolarization, Ca_V_1.2 channel closure, and vasorelaxation. We recently demonstrated that pregnancy-induced decreases in the myogenic tone of uterine arteries also involve the upregulation of RyR expression/function and enhanced Ca^2+^ sparks [271]. Moreover, pregnancy promotes the colocalization of RyR1/2 and the BK_Ca_ channel β1 subunit, leading to enhanced Ca^2+^ spark-STOC coupling [261]. The increased Ca^2+^ spark-STOC coupling then boosts STOCs, resulting in reduced uterine arterial myogenic tone in pregnancy [261,271].

NO and hydrogen sulfide (H_2_S) are recognized as important regulators of vascular function. Pregnancy increases NO and H_2_S production in both human and sheep uterine arteries, which contributes to estrogen-induced uterine vasodilation in pregnancy [244,272,273,274]. NO is a potent stimulator of the BK_Ca_ channel in VSMCs [139]. It is expected that NO also triggers BK_Ca_ activation in uterine arteries to promote vasodilation in pregnancy as there is a parallel increase in both the production of NO and cGMP and expression of the BK_Ca_ channel in uterine arteries during pregnancy [57,203,233]. A recent study reveals that H_2_S elicits vasodilation of uterine arteries via activating the BK_Ca_ channel [236].

EDHF plays an important role in regulating uterine vascular contractility during pregnancy [220,275]. Endothelial SK_Ca_2.3 and IK_Ca_ channels mediate endothelial membrane hyperpolarization and participate in EDHF-mediated vasodilator response [148,276]. Pregnancy significantly potentiates EDHF-mediated vasodilation of uterine arteries [204,277]. For example, EDHF contributes to ~30% of endothelium-dependent vasorelaxation of uterine arteries in nonpregnant rats and this fraction increases to ~70% in pregnant animals [277]. A combination of apamin plus charybdotoxin or TRAM 34, but not of apamin plus the BK_Ca_ channel blocker iberiotoxin, abolished the EDHF-mediated dilation of human and rat uterine arteries, suggesting that SK_Ca_ and IK_Ca_ channels are major mediators of EDHF responses in uterine arteries [204,275,278]. MEGJs provide direct contact between the ECs and VSMCs. MEGJs are the primary pathway of EDHF-mediated relaxation of myometrial arteries in pregnancy [148]. The SK_Ca_ channel may also mediate NO-induced relaxation of uterine arteries [279]. In addition, the SK_Ca_ channel in uterine VSMCs participates in regulating the myogenic tone of uterine arteries [68].

The SK_Ca_2.3 and IK_Ca_ channels also participate in uteroplacental angiogenesis and vascular remodeling during pregnancy. Inhibiting SK_Ca_2.3 and IK_Ca_ channels in HUVECs with apamin and TRAM 34, respectively, inhibits the secretion of angiogenic factors, proliferation/migration, and tube formation [280]. On the other hand, overexpression of the SK_Ca_2.3 channel increases the diameter of uterine arteries [281]. Similarly, SK_Ca_2.3 channel overexpression also increases the ratio of VEGF to sFlt-1 and vessel size/numbers in the placenta [282].

## 6. K_Ca_ Channels and Uteroplacental Circulation in Pregnancy Complications

### 6.1. Aberrant Expression/Function of Uteroplacental Vascular K_Ca_ in Pregnancy Complications

The expression of the BK_Ca_ channel β1 subunit is repressed in human placental chorionic plate arteries in preeclampsia, which is associated with impaired NO-induced vasodilation [69]. In addition, preeclampsia also reduces the expression of the BK_Ca_ channel β1 subunit in umbilical vein ECs [283]. In a sheep model of preeclampsia, it is found that high-altitude acclimatization downregulates the BK_Ca_ channel β1 subunit in uterine arteries leading to increased uterine vascular tone [234,284]. The expression of the BK_Ca_ channel β1 subunit is also downregulated in the uterine arteries of a mouse model of preeclampsia induced by electrical stimulation, leading to increased uteroplacental vascular resistance [285].

Both SK_Ca_ and IK_Ca_ channels are downregulated in human placental chorionic plate arteries in preeclampsia [241]. The IK_Ca_ channel is also downregulated in ECs of the umbilical artery and vein from preeclamptic pregnancy [238,283]. The contribution of MEGJs to EDHF-induced relaxation of myometrial arteries is diminished in preeclampsia [214]. Treating cultured HUVECs with plasma from preeclamptic women mimics the impacts of preeclampsia on IK_Ca_ channel expression [238]. An increase in circulating testosterone level is an important risk factor for preeclampsia [286,287,288]. In a rat model of preeclampsia/FGR, elevated levels of plasma testosterone result in FGR [237]. Uterine arteries from pregnant rats chronically treated with testosterone display augmented vasoconstriction to thromboxane, phenylephrine, and angiotensin II. In addition, the prolonged testosterone treatment also downregulates the SK_Ca_2.3 channel in uterine arteries, leading to diminished EDHF-mediated relaxation [237]. In pregnant guinea pigs, chronic hypoxia attenuates EDHF-mediated relaxation of uterine arteries [289], possibly due to impaired SK_Ca_/IK_Ca_ channel expression/function.

Gestational diabetes is associated with the downregulation of both BK_Ca_ channel α and β1 subunits in human umbilical arterial smooth muscle cells [290]. Using a rat model in which gestational diabetes is induced by the injection of streptozotocin during pregnancy, Gokina’s group demonstrates that EDHF-induced uteroplacental vasodilation is impaired owing to reduced basal and agonist-stimulated [Ca^2+^]_i_ in ECs [291]. Moreover, they also provide evidence that diabetes selectively causes dysfunction of the IK_Ca_ channel in uteroplacental arteries, which attributes to the impaired EDHF response [292,293]. Likewise, EDHF-induced vasorelaxation is reduced in uterine arteries of streptozotocin-treated pregnant mice [294].

### 6.2. Mechanisms Underlying the Dysregulation of K_Ca_ Channels in the Uteroplacental Circulation

#### 6.2.1. Hypoxia and HIFs

Hypoxia during gestation is a major insult to maternal cardiovascular homeostasis and complicates adaptive changes in the uteroplacental circulation [295,296]. HIFs play a crucial role in cellular (mal)adaptation in response to hypoxia. Levels of HIF-1α increase in preeclamptic placentas, in placentas from human high-altitude pregnancy, in uterine arteries of high-altitude acclimatized pregnant sheep, and in placentas of a hypoxic rodent model of preeclampsia [297,298,299,300]. There are complex interplays among HIFs, ROS/endoplasmic reticulum (ER) stress, and epigenetic regulation [296]. For example, HIF-1α is stabilized by mitochondrial ROS [301], whereas HIF-1α through miR-210-induced downregulation of ISCU promotes mitochondrial ROS production [302]. Moreover, DNMT expression is upregulated by HIF-1α [303]. These factors can act alone and in concert to contribute to the pathogenesis of preeclampsia.

Gestational hypoxia attenuates the pregnancy-induced rise in uteroplacental blood flow, leading to increased incidence of preeclampsia and IUGR [299,304,305,306,307]. K_Ca_ channels in vascular beds are major targets of hypoxia [37,308]. Gestational hypoxia directly downregulates the BK_Ca_ channel β1 subunit and suppresses the upregulation of the BK_Ca_ channel β1 subunit and SK_Ca_ channels in ovine uterine arteries during pregnancy [68,234]. The attenuated expression of K_Ca_ channels culminates in decreased channel activities, leading to increased myogenic tone and diminished K_Ca_ channel-mediated vasorelaxation.

#### 6.2.2. Epigenetic Regulation

MicroRNAs (miRs) are non-coding RNAs and play important roles in regulating gene expression. miRs regulate gene expression by interacting with the 3′-untranslated region (3′-UTR) of target mRNAs to induce mRNA degradation and translational repression [309]. Circulating and uteroplacental levels of miR-210, a target of HIF-1α, are increased in preeclampsia, in high-altitude pregnancy, and in a high-altitude hypoxic sheep model of preeclampsia [284,310,311,312,313]. KCNMB1 and RYR2 each contain a miR-210 complementary binding site in their 3′-UTRs and both of them are targets of miR-210 [313]. Indeed, gestational hypoxia via miR-210-mediated downregulation of RyR2 and BK_Ca_ channel β1 subunit disrupts the Ca^2+^ spark-STOC coupling in uterine arteries and hence increases uterine arterial myogenic tone [313].

The dynamic of DNA methylation and demethylation is also an important epigenetic mechanism to fine-tune gene expression. DNA methylation catalyzed by a family of DNMTs transfers a methyl group from S-adenyl methionine to the cytosine residue in a CpG dinucleotide(s) to form 5-methylcytosine (5mC). In general, methylation in the promoter regions of genes is associated with the repression of transcription [314]. On the other hand, active DNA demethylation is initiated by TETs which mediate the oxidation of 5mC to 5-hydroxymethylcytosine (5hmC), thus reviving gene transcription [315]. Gestational hypoxia is found to upregulate DNMT3b in uterine arteries, hence enhancing DNA methylation [316] (Figure 3). TET1 is also a target of miR-210 and gestational hypoxia via miR-210 triggers the downregulation of TET1 in uterine arteries [284,317]. TET1 deficiency nullifies pregnancy-induced DNA demethylation [235,284,317]. Overall, these changes lead to hypermethylation of KCNMB1, downregulation of the BK_Ca_ channel β1 subunit in uterine arteries, and increased myogenic tone [284,316,317]. Gestational hypoxia also suppresses the expression of ERα in uterine arteries through hypermethylating the Erα-encoding gene ESR1, which could in turn impairs pregnancy- and estrogen-induced BK_Ca_ channel β1 subunit upregulation [318,319,320].

#### 6.2.3. Oxidative/ER Stress

Pregnancy complications are in a state of exaggerated oxidative stress [321]. Reactive oxygen species (ROS) have been implicated in the pathogenesis of various cardiovascular disorders. Mitochondria and NADPH oxidases (NOX) are major sources of ROS in the vasculature [322]. Preeclampsia and gestational hypoxia are found to increase the expression/activity of NOX2 and ROS in the uterine arteries of pregnant sheep and HUVECs [283,300]. Mitochondrial ROS are increased in the placenta of a rat model of preeclampsia produced by reduced uterine perfusion pressure [323]. Likewise, gestational hypoxia also increases mitochondrial ROS via miR-210-mediated downregulation of ISCU and subsequent perturbation of mitochondrial respiration in uterine arteries [324]. ROS could exert its impacts on K_Ca_ channels directly or indirectly. Cys911 oxidation in the BK_Ca_ channel α subunit decreases Ca^2+^ sensitivity and impairs channel function [325]. Acute inhibition of ROS with apocynin (a NOX inhibitor) or N-acetylcysteine/EUK-134 (antioxidants) increases BK_Ca_ channel activity in uterine arterial VSMCs of pregnant sheep experiencing gestational hypoxia [259,300,326], suggesting that the BK_Ca_ channel in uterine arteries is tonically inhibited by ROS under hypoxia. Moreover, antioxidant treatment with N-acetylcysteine in ex vivo studies restores the capacity of estrogen to stimulate molecular and functional expression of the BK_Ca_ channel β1 subunit [259,326]. These findings suggest that gestational hypoxia-induced oxidative stress also impairs BK_Ca_ channel function by suppressing estrogen-induced KCNMB1 expression in uterine arteries. The Ca^2+^ spark-STOC coupling is disrupted by mitochondrial ROS, leading to increased myogenic tone. ROS derived from NOX2 also repress the expression of the BK_Ca_ channel β1 subunit in HUVECs from preeclamptic pregnancy [283]. Impaired uteroplacental perfusion in mice with gestational diabetes is associated with elevated oxidative stress in uterine arteries [224]. Although the impact of ROS on BK_Ca_ channel expression/function is not examined in uteroplacental VSMCs of gestational diabetes, NOX-derived ROS have been shown to mediate the downregulation of the BK_Ca_ channel β1 subunit in VSMCs of other vascular beds in diabetic mice [327].

The expression of the SK_Ca_ channel is downregulated by NOX2-derived ROS in umbilical vessels and HUVECs from preeclamptic pregnancy [238,283]. This downregulation is imitated by treating HUVECs with serum from women with preeclampsia, oxidized low-density lipoprotein, palmitic acid, and the superoxide donor xanthine/xanthine oxidase mixture [238,328]. Similarly, exogenous H_2_O_2_ suppresses the expression of IK_Ca_ and/or SK_Ca_ channels in cultured HUVECs [329]. In human uterine microvascular ECs, NOX4-derived superoxide mediates the downregulation of K_Ca_2.3 and K_Ca_3.1 channels induced by serum from preeclamptic women [246]. In addition, NOX4-derived ROS also promote the internalization of K_Ca_2.3 and K_Ca_3.1 channels by increasing the association of these channels with caveolin-1, clathrin, and Rab5c in human uterine microvascular ECs [250]. Testosterone suppresses mitochondrial respiration in uteroplacental and vascular cells [330,331]. Thus, the downregulation of the SK_Ca_ channel in uterine arteries of pregnant rats chronically treated with testosterone is probably mediated by mitochondrial ROS [237]. Chronic administration of Mito-Tempo in diabetic mice also normalizes the impaired SK_Ca_ activity in heart ECs [332].

Endoplasmic reticulum (ER) stress occurs when ER homeostasis is perturbed. Placentas from preeclamptic pregnancy, FGR, and diabetic pregnancy undergo ER stress [333,334,335,336]. Gestational hypoxia also triggers ER stress and activates unfolded protein response (UPR) in the human placenta and in ovine uterine arteries [337,338]. The ER stress inhibitor tauroursodeoxycholic acid and PERK inhibitor GSK2606414 relieve hypoxia-mediated suppression of Ca^2+^ sparks/STOCs and decrease myogenic tone in uterine arteries [337]. ER stress is found to cause downregulation of the BK_Ca_ channel β1 subunit and suppression of BK_Ca_ channel activity in VSMCs [339]. Similarly, SK_Ca_2.3 and IK_Ca_ channel activities are also suppressed by ER stress in ECs [340]. Thus, ER stress also contributes to the maladaptation of the uteroplacental circulation by impairing K_Ca_ expression/function in pregnancy complications.

#### 6.2.4. PKC

Preeclamptic serum increases PKC signaling in cultured HUVECs [341,342]. Gestational hypoxia upregulates PKC in the uterine arteries of pregnant sheep [343]. Activation of PKC inhibits BK_Ca_ channel activity and increases myogenic tone in the uterine arteries of pregnant sheep [233]. This mechanism also contributes to gestational hypoxia-induced suppression of SK_Ca_ channel activity [262]. Peroxisome proliferator-activated receptor-γ (PPARγ), a ligand-activated transcription factor, has been implicated in the pathogenesis of preeclampsia [344]. Mesenteric arteries from transgenic mice expressing dominant-negative mutant PPARγ displays increased myogenic tone, due to PKC-mediated inhibition of the BK_Ca_ channel in VSMCs [345]. Similarly, chronic inhibition of PPARγ during rat pregnancy attenuates uterine vasodilation and causes FGR [346]. Moreover, elevated expression of PKCβ in diabetic mouse aortas promotes the BK_Ca_ channel β1 subunit downregulation by impairing AKT signaling [327].

## 7. Conclusions

Uteroplacental blood flow increases markedly in pregnancy to meet the demand for placental and fetal growth. Uteroplacental vessels undergo extensive structural and functional changes to accommodate increased uteroplacental perfusion. However, these adaptative changes are impaired in pregnancy complications. Precise mechanisms underlying the adaptation/maladaptation of the uteroplacental circulation are not completely understood. Findings over the past twenty years have suggested important roles of K_Ca_ channels in the regulation of the uteroplacental circulation under physiological and pathophysiological conditions. Notably, estrogen plays a central role in upregulating K_Ca_ channel expression/function leading to reduced uterine vascular tone in normal pregnancy. Lines of evidence suggest that multiple mechanisms including HIFs/miR-210, oxidative stress/ER stress, and PKC contribute to K_Ca_ channel dysfunction in uteroplacental vessels, resulting in the maladaptation of the uteroplacental circulation in pregnancy complications. Thus, restoring K_Ca_ channel expression/function by targeting HIFs/miR-210, oxidative stress/ER stress, and PKC may offer avenues for the development of therapeutics for pregnancy complications.

## Data Availability

Data are contained within the article.
